# Supplementary figures and images for: Anti-BIRC5 autoantibody serves as a valuable biomarker for diagnosing AFP-negative hepatocellular carcinoma
Source: PeerJ. 2024 May 31;12:e17494. doi: 10.7717/peerj.17494 (PMC11146321; doi:10.7717/peerj.17494)

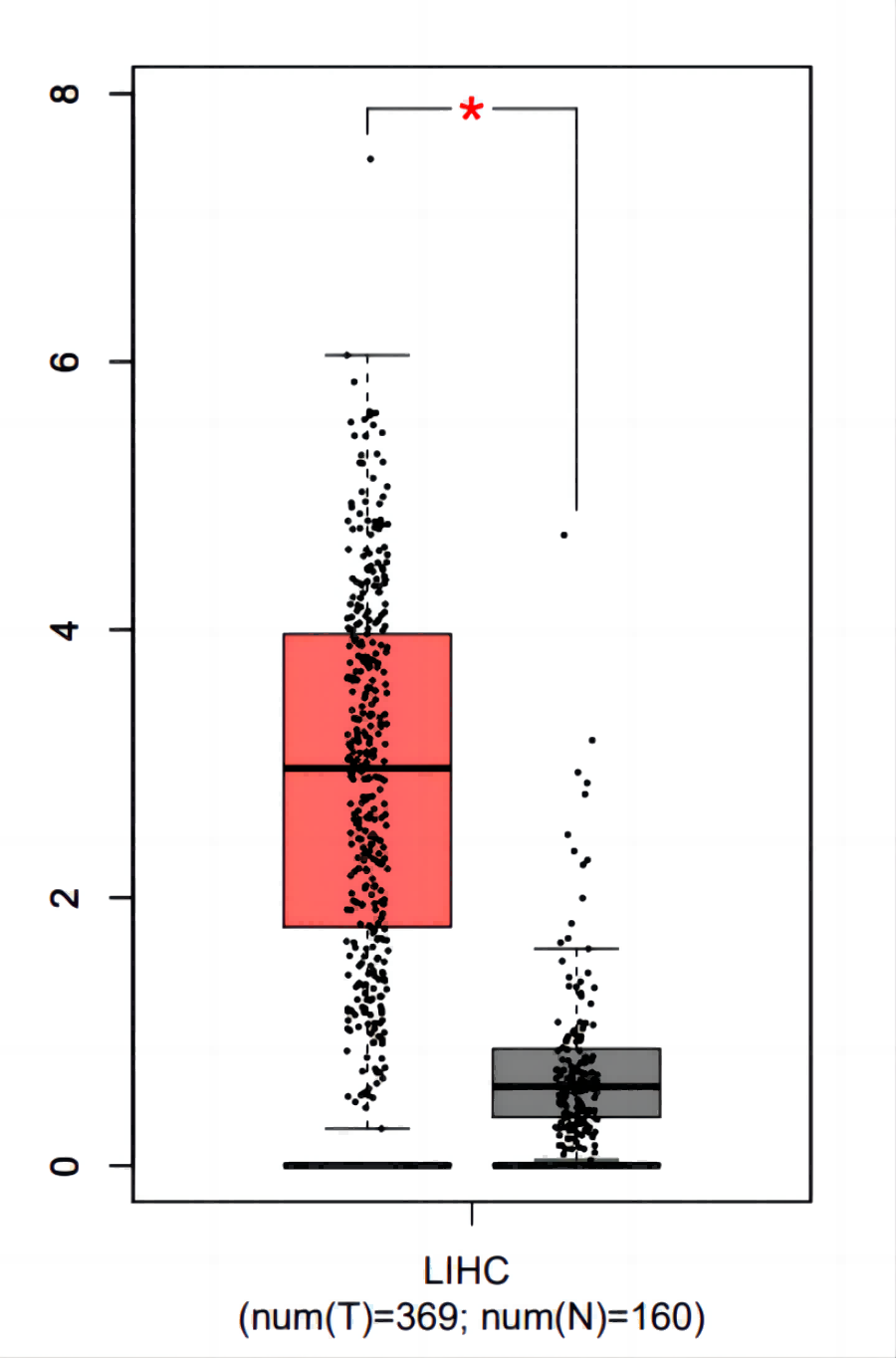

Supplement: Supplemental Information 2 [file peerj-12-17494-s002.png]
