# Supplementary material for: Anti-BIRC5 autoantibody serves as a valuable biomarker for diagnosing AFP-negative hepatocellular carcinoma
Source: PeerJ. 2024 May 31;12:e17494. doi: 10.7717/peerj.17494 (PMC11146321; doi:10.7717/peerj.17494)
Supplement: Supplemental Information 3 [file peerj-12-17494-s003.docx]

**Miame Checklist**

**Part 1 Experiment description**

The type of the experiment :such as normal-versus-diseased comparison

experimental variables:The relative titer of the antibody.The relative content of antibody to be measured is reflected by fluorescence signal.

The customized protein chip contains 154 proteins or protein fragments containing GST labels purchased from CDI laboratories in the United States. Among the 154 proteins, There were 143 protein coded by cancer-driven gene, and the other 11 protein showed good performance in the previous verification of our research group (CIP2A/p90, RalA, IMP1, IMP2, IMP3, Two segments of CyclinBl, c-Myc, YWHAZ, RBM39, and Survivin).

The antigen fixed on the protein chip and the tested antibody in the blood serum developed a specific binding, and after adding the anti-human IgG nonspecifically binding to antigen-antibody complexes.The relative content of antibody to be measured is reflected by fluorescence signal.

**Part 2 Array design**

The arrangement of protein chips is shown in the figure.(Survin is also called BIRC5)


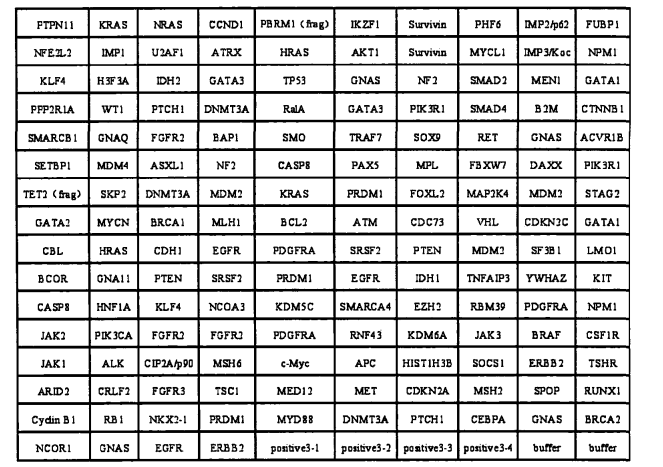
Array type :human

**Part 3 Samples**

All samples were derived from human serum.All cases of Hepatocellular carcinoma were first diagnosed by ultrasound, CT, MRI and other imaging methods in accordance with Chinese norms for diagnosis and treatment of hepatocellular carcinoma, without any operation or radiotherapy or chemotherapy treatment, and were confirmed by pathological diagnosis at a later stage. Health were obtained from the same period of physical examination population, excluding abnormal liver function, liver disease history or other tumor history. All subjects need to sign informed consent and be approved by the Ethics Committee of Zhengzhou University.

**Part 4 Hybridizations**

1.Preparatory

Sealing fluid, cleaning fluid,Serum and serum diluent diluted at 1:50,Chip rewarming (first taken from -80 ℃, balanced at 4 ℃ 30 min, then balanced at room temperature15min ).

1. Sealing

Add 200 ul of sealing liquid to each bloc k and seal at room temperature at 20 rpm on the side shaking bed for 3 h.

1. Primary antibody incubation

Discard the sealing fluid, add 200 diluted serum per block, Side swing table 20 rpm, 4 ℃ overnight.

1. Cleaning

The serum sample is sucked away, the enclosure is removed, the care is taken to avoid cross-contamination between the serum, and the core piece is placed in the core piece cleaning box containing PB ST cleaning solution. Wash 3 times on a horizontal shaker at 80 rpm, 10 mi n/times, room temperature.

1. Secondary antibody incubation

The core was transferred to the secondary antibody incubation box (3 ml/box), the lateral shaking table was 40 rpm, and the incubation was conducted for 1 h at room temperature and away from light.

1. Cleaning

After washing 3 times with the method in step (3) above, replace PBST washing liquid with ddH20 and wash 2 times, each time 10 min.

1. Drying

The chip is placed in the core dryer to dry the core (note that the chip can not touch or scratch the surface of the chip during the process of taking the chip)

**Part 5 Measurements**

To read the data, the coreLuxScan 10K microarray chip scanner is operated according to the standard and the specification.Signal for picking up IgG antibody F532 Median (532 channel front view value).Calculate the ratio between the front and back values of each protein, that is, F median/B median. The average value of Fmedian/B median of two complex holes in the same protein is defined as SNR value (signal-to-noise ratio) and then analyzed.

**Part 6 Normalization controls**

1. Quality inspection of custom protein chip

After the chip was prepared, the quality of the chip was detected by anti-GST antibody. 1) The correlation of the SNR produced by the anti-GST cross result with two multiple holes on the same prptein on the core chip, R^2^ was 0. 960, indicating that the overall repeatability of the chip repeatability is good. 2) The detection rate of protein was evaluated by the signal-to-noise ratio of negative control , and the results showed that the detection rate of protein was above 99%, indicating that the chip was qualified.

1. Stability analysis of protein chip detection

In order to detect the stability of the chip, the same serum was randomly selected to carry out the re-measurement analysis of the chip, the detection time, and the position on the core chip. A total of 30 times were re-repeated.As shown in the following picture,draw the duplicate matrix graph 10 times at random. The lower left is the scatter-plot after linear correlation fitting, and the upper right is the R^2^ value of linear correlation analysis (* * * means.*P* <0.001), where the middle is the cumulative density distribution of each protein SNR value in a single sample.


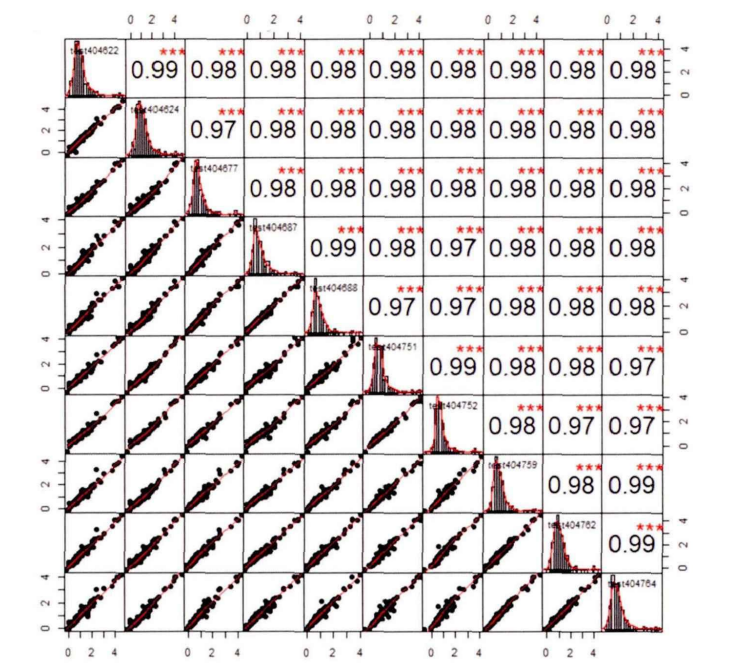


1. Data preprocessing

The difference of the background value of each egg white in the serum of each case was analyzed and observed, which was shown by the coefficient of variation (CV value). The C and V values of 98.67% of serum background value were within 15%, which was considered that the background value of different protein in the same serum were more uniform, and the background value of the serum could be shown by using the average value**.**Set the threshold (Mean + 2SD) to eliminate samples whose background value is larger than this threshold.
